# Use of Biologics During Pregnancy Among Patients With Autoimmune Conditions

**DOI:** 10.1001/jamanetworkopen.2025.10504

**Published:** 2025-05-15

**Authors:** Celeste L. Y. Ewig, Yanning Wang, Nicole E. Smolinski, Thuy Nhu Thai, Sonja A. Rasmussen, Almut G. Winterstein

**Affiliations:** 1 Department of Pharmaceutical Outcomes and Policy, College of Pharmacy, University of Florida, Gainesville; 2 Center for Drug Evaluation and Safety, University of Florida, Gainesville; 3 Department of Population Medicine, Harvard Medical School and Harvard Pilgrim Health Care Institute, Boston, Massachusetts; 4 Department of Genetic Medicine, Johns Hopkins University School of Medicine, Baltimore, Maryland; 5 Department of Gynecology and Obstetrics, Johns Hopkins University School of Medicine, Baltimore, Maryland

## Abstract

**Importance:**

Continuation of biologics in patients with an autoimmune condition who become pregnant involves weighing consequences of pregnancy-related changes in disease severity and potential teratogenic effects of medications. Characterization of biologic treatment patterns during pregnancy may provide insight into maternal and fetal risks and benefits.

**Objective:**

To describe the utilization pattern of biologics in pregnant individuals with autoimmune conditions.

**Design, Setting, and Participants:**

This cohort study used data from Merative MarketScan Research Databases, which contain administrative claims of commercially insured individuals in the US. Pregnant patients aged 16 to 55 years with an autoimmune condition and biologic use 6 months before conception between January 1, 2011, and December 31, 2022, were included. The data were analyzed between October 15, 2024, and February 28, 2025.

**Exposure:**

Use of biologics for autoimmune disease after conception.

**Main Outcomes and Measures:**

The proportion of patients who used biologics for Crohn disease, ulcerative colitis, psoriasis or psoriatic arthritis, rheumatoid arthritis, ankylosing spondylitis, systemic lupus erythematosus, and multiple sclerosis was assessed, and the association between underlying autoimmune disease and use of biologics during pregnancy was measured using multivariable logistic regression.

**Results:**

A total of 6131 pregnant patients (median [IQR] age, 32 [29-36] years) with an autoimmune condition were included. The most prevalent conditions were Crohn disease (1372 patients [25.6%]) and rheumatoid arthritis (1295 patients [24.1%]). Of all patients, 4393 (71.6%; 95% CI, 70.5%-72.8%) used biologics at least once during pregnancy. Among pregnancies with live birth outcomes, biologic use declined throughout gestation, with 2981 patients (68.6% [95% CI, 67.2%-70.0%]), 2555 patients (58.8% [95% CI, 57.3%-60.3%]), and 2113 patients (48.6% [95% CI, 47.1%-50.1%]) using biologics during the first, second, and third trimesters, respectively, and 3350 patients (77.1% [95% CI, 75.8%-78.3%]) using them post partum. Compared with pregnant patients with rheumatoid arthritis, those with Crohn disease (odds ratio [OR], 7.88 [95% CI, 5.93-10.47]) and ulcerative colitis (OR, 5.35 [95% CI, 3.73-7.66]) were more likely to use biologics, while those with psoriasis or psoriatic arthritis (OR, 0.65 [95% CI, 0.52-0.80]) were less likely.

**Conclusions and Relevance:**

In this cohort study, a decline in the use of biologics for autoimmune disease was observed during the pregnancy period that rebounded only partially thereafter. Notable variations in use across autoimmune conditions suggest that indication-specific risk-benefit assessments of biologic use are needed.

## Key Points

**Question:**

Does use of biologics for 7 diverse autoimmune conditions change during pregnancy?

**Findings:**

In this cohort study of 6131 individuals with an autoimmune condition and prepregnancy (prior 6 months) biologic use, 71.6% continued use at least once during pregnancy. Individuals with Crohn disease and ulcerative colitis were more likely to continue use during pregnancy compared with individuals with rheumatoid arthritis, while patients with psoriasis or psoriatic arthritis were less likely to continue.

**Meaning:**

The use of biologics during pregnancy is associated with the underlying autoimmune condition, suggesting perceived or true variability in risks and benefits.

## Teaser

This cohort study describes the utilization patterns of biologics among pregnant patients with autoimmune conditions from preconception to post partum.

## Introduction

An estimated 2% of women of childbearing age^1^ and 4.1% of pregnant individuals^2^ have an autoimmune condition. Management of autoimmune disease in pregnant populations is challenging due to the need to optimize treatment risks and benefits to both mother and fetus. Medications widely used by patients with autoimmune disease are also associated with miscarriage and major congenital anomalies^3,4^ and are recommended to be discontinued several weeks prior to conception.^5^ On the other hand, active disease during pregnancy is associated with preterm delivery,^6,7^ small-for-gestational age infants,^6,7^ and stillbirth.^7^ For many autoimmune conditions, newer treatment options, biologic anti-inflammatory, or immunomodulating agents (biologics) are recommended as alternatives to traditional disease-modifying agents in patients with moderate to severe conditions. The success of these biologics in achieving disease remission has led to an increasing trend in their use in the overall population of people with autoimmune disease,^8,9^ as well as in pregnant individuals,^10,11,12^ with previous studies reporting biologic use in 5.7% of pregnant patients with an autoimmune condition.^12^ However, evidence on biologic utilization patterns during pregnancy is incomplete. Most studies focused broadly on exposure during pregnancy in individuals with similar indications; were not limited to patients with an established benefit for biologic use before conception (ie, as reflected from prepregnancy use); or provided limited information on postpartum use, which may provide a more complete description of overall use by an individual with an autoimmune condition.

Despite the increase in biologic use among pregnant patients, evidence supporting the safe use of biologics during pregnancy is limited. Most studies have focused on earlier approved biologics, such as tumor necrosis factor (TNF) inhibitors^13,14,15,16^; pregnancies ending in live births^17,18^; or infant rather than maternal outcomes.^14,16^ Studies evaluating newer biologics, such as tocilizumab^19,20^ and vedolizumab,^20,21^ in pregnant populations are limited, leading to inconsistent recommendations regarding use of biologics during pregnancy.^22,23,24,25^ The complexity of the issue is compounded by the heterogeneous effect of pregnancy on the underlying autoimmune disease. Autoimmune conditions that are driven by T-helper cell 1 and 17, such as rheumatoid arthritis and multiple sclerosis, have been shown to improve during pregnancy, while those dominated by effects of T-helper cell 2 pathways, such as systemic lupus erythematosus, tend to worsen.^26^

To understand how these factors are associated with treatment decisions during pregnancy, this study aimed to describe the patterns of biologic continuation during and after pregnancy across patients with varying types of autoimmune conditions. We focused on individuals with biologic use prior to conception, in whom the benefit of biologics for their underlying condition had already been established, to capture pregnancy-specific risk-benefit decisions made by clinicians and patients.

## Methods

### Study Design

This retrospective cohort study was conducted using Merative MarketScan Commercial Claims Research Databases. These databases provide patient-level data on enrollment and health care use for a national sample of privately insured individuals in the US. The study was reviewed by the University of Florida Institutional Review Board, and the requirement for written informed consent was waived due to the deidentified nature of the data. This study complied with the Strengthening the Reporting of Observational Studies in Epidemiology (STROBE) reporting guideline.^27^

Information available within the claims research databases includes an individual’s diagnoses and procedures associated with inpatient and outpatient health care visits. Details of medication use are available within the pharmacy dispensing claims. For this study, we used data from January 1, 2010, through December 31, 2022.

### Study Population

Pregnant women aged 16 to 55 years with conception between January 1, 2011, and December 31, 2021, and an autoimmune condition were eligible for study inclusion. We used a previously validated algorithm to capture pregnancy episodes from administrative claims data. The algorithm uses diagnosis and procedure codes for medical encounters to identify and characterize pregnancy outcomes and retrospectively estimate conception date.^28,29,30,31^ Pregnant patients were required to have a minimum of 6 months continuous health care coverage prior to the date of conception through at least 6 months after the pregnancy end date. Individuals could contribute more than 1 pregnancy as long as all criteria for inclusion and exclusion were met. All pregnancies, regardless of a live birth and non–live birth outcome, were considered.

We selected 7 autoimmune conditions for inclusion a priori based on their prevalence among pregnant patients and availability of biologic treatments.^1^ These conditions include Crohn disease, ulcerative colitis, rheumatoid arthritis, psoriasis or psoriatic arthritis, ankylosing spondylitis, systemic lupus erythematosus, and multiple sclerosis. Individuals with more than 1 documented autoimmune condition were categorized as having multiple conditions. Pregnant patients were considered to have an autoimmune condition if they had a relevant diagnosis code based on *International Classification of Diseases, Ninth Revision, Clinical Modification* or *International Statistical Classification of Diseases, Tenth Revision, Clinical Modification* diagnosis codes (eTable 1 in Supplement 1) documented during any health care visit within 6 months before conception or during the pregnancy period. Among these pregnancies, we required biologic use during the 180-day preconception window.^11,12^ Use of biologics indicated for an autoimmune condition (eTable 2 in Supplement 1) was identified using the National Drug Code from pharmacy dispensing claims and *Current Procedural Terminology* or Healthcare Common Procedure Coding System codes indicating biologic administration during a health care visit. The dispensing or visit date was assumed to be the date of biologic use. Since our study aimed to differentiate between prepregnancy use and (intended) continuation during pregnancy, further categorized by trimester, residual supply from previous prescription fills, or extended medication half-lives were not considered.

### Outcome Measure

Our primary outcome was the proportion of qualifying pregnancies with biologic use after conception. We defined use by the presence of a dispensing or administration claim for a unique biologic during the relevant gestation period. Biologic use was assessed across 5 periods anchored from the conception date: preconception (period from 180 days to 1 day prior to conception), first trimester (day 1 to day 83 after conception), second trimester (day 84 to day 181 after conception), third trimester (day 182 after conception to pregnancy end date), and post partum (pregnancy end date plus 180 days). We further examined biologic use stratified by therapeutic category during each pregnancy period, as well as temporal trends in biologic use among pregnant patients with an autoimmune condition during the study period.

### Statistical Analysis

Descriptive assessments of biologic use across trimesters were limited to live births, while all other analyses considered pregnancies with both live and non–live birth outcomes. To examine factors associated with biologic use after conception, we modeled biologic use as our dependent variable using multivariable logistic regression with generalized estimating equations to account for correlations among women with multiple pregnancy episodes during the study period. Covariates included in our regression model were maternal age at conception, year of conception, underlying autoimmune diagnosis, and use of systemic glucocorticoids during the 6 months leading up to conception. Median maternal age was selected as our reference group to examine the effects of age on our outcome of interest. Information regarding race and ethnicity were not available to be collected from our data source. We selected rheumatoid arthritis as our referent condition due to its high prevalence and sufficient proportions of patients with and without biologic use to provide more robust estimates. We adjusted for gestational age at pregnancy end to address potential differences in pregnancy length. Separate analyses were performed for pregnancies with live and non–live birth outcomes. The Cochran-Armitage trend test was used to evaluate the annual proportion of pregnant patients who used a biologic from 2011 to 2021.^12^ All data processing and statistical analyses were performed between October 15, 2024, and February 28, 2025, using SAS, version 9.4 (SAS Institute Inc). The threshold for statistical significance was set at *P* < .05.

## Results

### Study Population

We identified 89 169 pregnancies among women aged 16 to 55 years with an autoimmune diagnosis of interest and who met our inclusion criteria. Of these patients, 6131 (6.9%) had recent biologic use within 6 months prior to conception and were included in our study (median [IQR] age, 32 [29-36] years). The most prevalent autoimmune conditions were Crohn disease (1372 patients [25.6%]) and rheumatoid arthritis (1295 patients [24.1%]), with 1386 patients (22.6%) having more than 1 concurrent autoimmune diagnosis during pregnancy. Of the pregnancies included in the study, 4342 (70.8%) resulted in a live birth, 1319 (21.5%) resulted in spontaneous abortion, and 316 (5.2%) were induced abortions. A history of system glucocorticoid use 6 months before conception was observed in 1571 patients (25.6%) (Table).

**Table.** Characteristics of Patients With an Autoimmune Condition and Biologic Use During Pregnancy

Characteristic	Patients, No. (%)		
	All pregnancies	Use during pregnancy	No use during pregnancy
Patients, No. (%) [95% CI]	6131	4393 (71.6) [70.5-72.8]	1738 (28.4) [27.2-29.5]
Maternal age, y			
Median (IQR)	32 (29-36)	32.0 (29-35)	33.0 (29-36)
Age group			
16-19	42 (0.7)	31 (0.7)	11 (0.6)
20-24	412 (6.7)	304 (6.9)	108 (6.2)
25-29	1253 (20.4)	883 (20.1)	370 (21.3)
30-34	2147 (35.0)	1503 (34.2)	644 (37.1)
35-39	1635 (26.7)	1186 (27.0)	449 (25.8)
40-44	562 (9.2)	425 (9.7)	137 (7.9)
≥45	80 (1.3)	61 (1.4)	19 (1.1)
Autoimmune condition			
Crohn disease	1372 (22.4)	1226 (27.9)	146 (8.4)
Rheumatoid arthritis	1295 (21.1)	788 (17.9)	507 (29.2)
Ulcerative colitis	945 (15.4)	519 (11.8)	426 (24.5)
Psoriasis or psoriatic arthritis	608 (9.9)	535 (12.2)	73 (4.2)
Multiple sclerosis	261 (4.3)	92 (2.1)	169 (9.7)
Ankylosing spondylitis	184 (3.0)	116 (2.6)	68 (3.9)
Systemic lupus erythematosus	80 (1.3)	39 (0.9)	41 (2.4)
Multiple conditions	1386 (22.6)	1078 (24.5)	308 (17.7)
No. of autoimmune conditions			
1	4745 (77.4)	3315 (75.5)	1430 (82.3)
2-3	1374 (22.4)	1071 (24.4)	303 (17.4)
4-5	12 (0.2)	7 (0.2)	5 (0.3)
Use of systemic glucocorticoids			
6 mo Before conception	1571 (25.6)	1132 (25.8)	439 (25.3)
During pregnancy	933 (15.2)	685 (15.6)	248 (14.3)
Pregnancy outcomes			
Live birth outcome	4342 (70.8)	3194 (72.7)	1148 (66.1)
Term delivery	3718 (60.6)	2759 (62.8)	959 (55.2)
Preterm delivery	624 (10.2)	435 (9.9)	189 (10.9)
Non–live birth outcome	1789 (29.2)	1199 (27.3)	590 (33.9)
Spontaneous abortion	1319 (21.5)	875 (19.9)	444 (25.5)
Induced abortion	316 (5.2)	218 (5.0)	98 (5.6)
Ectopic pregnancy	118 (1.9)	78 (1.8)	40 (2.3)
Stillbirth	35 (0.6)	27 (0.6)	8 (0.5)
Mixed delivery^a^	1 (<0.1)	1 (<0.1)	0

^a^ Pregnancy with 1 live birth outcome and 1 non–live birth outcome.

### Use of Biologics During and After Pregnancy

**Figure 1.**
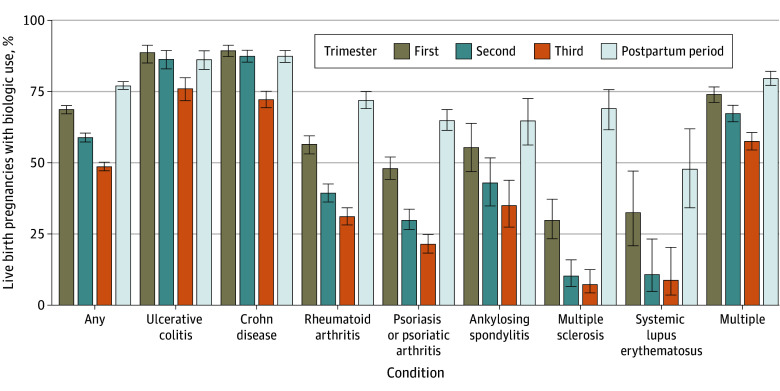
Prevalence of Biologic Use During and After Pregnancy Among Patients With Live Births Pregnant patients were required to have biologic use 6 months prior to conception; therefore, biologic use before the first trimester was 100%. Error bars indicate Wilson 95% CIs.

We identified 4393 pregnant patients (71.6% [95% CI, 70.5%-72.8%]) with biologic use at least once during the gestation period. Of the 1738 patients with no biologic use during pregnancy, 917 (52.8% [95% CI, 50.4%-55.1%]) resumed biologic use within 6 months after pregnancy end. Among pregnancies with live birth outcomes, biologic use declined consistently throughout gestation, with 2981 patients (68.6% [95% CI, 67.2%-70.0%]) using biologics in the first trimester, 2555 (58.8% [95% CI, 57.3%-60.3%]) in the second trimester, and 2113 (48.6% [95% CI, 47.1%-50.1%]) in the third trimester. Postpartum use rebounded in these patients but remained lower compared with the prenatal period (3350 patients [77.1%; 95% CI, 75.8%-78.3%]). The observed decline in use during pregnancy and the postpregnancy rebound were consistent across indications, although the magnitude of decline varied. The largest reduction in biologic use during pregnancy was noted among patients with multiple sclerosis (first trimester, 29.7% [95% CI, 23.2%-37.0%]; second trimester, 10.3% [95% CI, 6.5%-15.8%]; third trimester, 7.3% [95% CI, 4.2%-12.2%]) and systemic lupus erythematosus (first trimester, 32.6% [95% CI, 20.8%-47.0%]; second trimester, 10.9% [95% CI, 4.7%-23.0%]; third trimester, 8.7% [95% CI, 3.4%-20.3%]), while patients with ulcerative colitis (first trimester, 88.6% [95% CI, 85.1%-91.2%]; second trimester, 86.6% [95% CI, 85.3%-89.4%]; third trimester, 76.1% [95% CI, 71.9%-79.9%]) and Crohn disease (first trimester, 89.4% [95% CI, 87.3%-91.1%]; second trimester, 87.6% [95% CI, 85.3%-89.4%]; third trimester, 72.3% [95% CI, 69.3%-74.9%]) had the highest retention of biologic use (Figure 1; eTable 3 in Supplement 1).

Our study population had 6894 occurrences of unique biologic use in the prepregnancy period. When considering use of biologics after conception by therapeutic class, TNF inhibitors consistently remained the most frequent class of biologic agents used during pregnancy (first trimester, 3792 patients [80.5%]; second trimester, 2492 patients [84.4%]; third trimester, 2020 patients [83.5%]) and thereafter (post partum, 4360 [83.4%]) (eTable 4 in Supplement 1). Distribution of therapeutic classification of biologics used during pregnancy are shown in eFigure 1 in Supplement 1.

### Factors Associated With Biologic Use During Pregnancy

**Figure 2.**
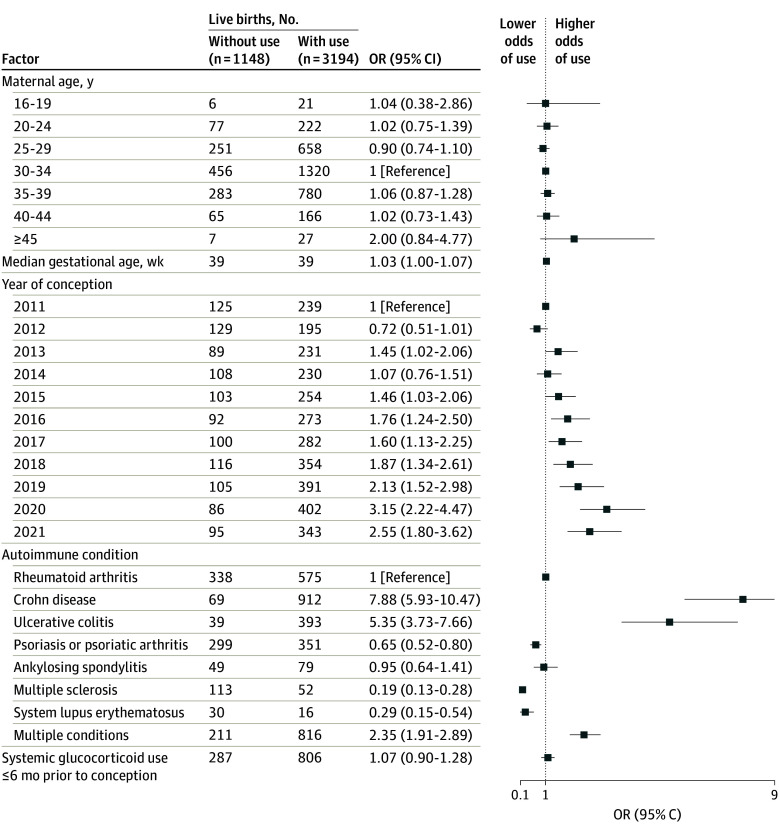
Association Between Autoimmune Condition and Biologic Use During Pregnancy (Live Birth Outcomes) OR indicates odds ratio.

**Figure 3.**
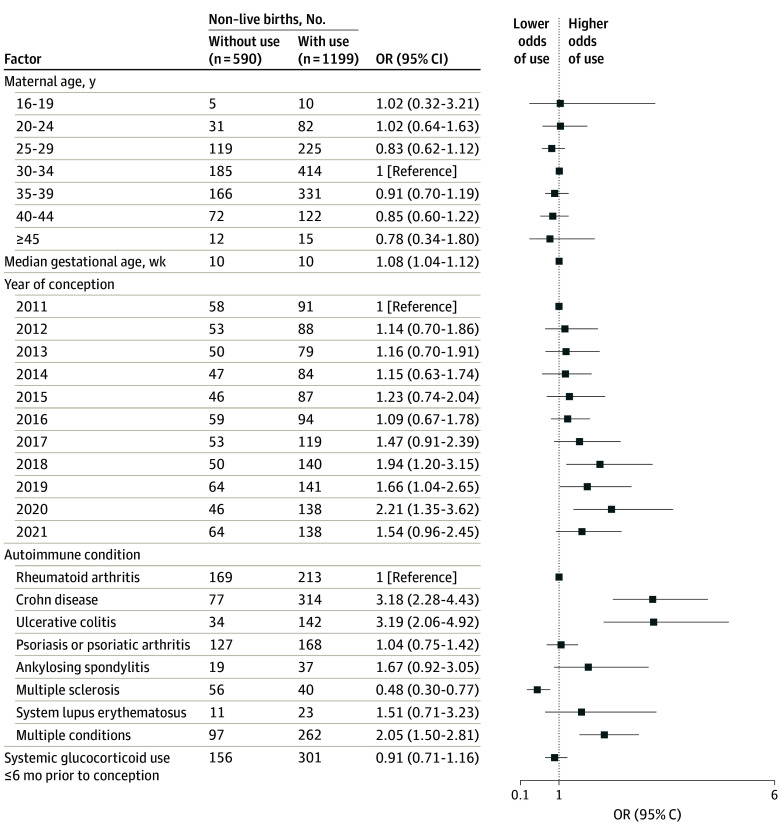
Association Between Autoimmune Condition and Biologic Use During Pregnancy (Non–Live Birth Outcomes) OR indicates odds ratio.

In pregnancies with live birth outcomes, patients with Crohn disease (odds ratio [OR], 7.88 [95% CI, 5.93-10.47]), ulcerative colitis (OR, 5.35 [95% CI, 3.73-7.66]), or multiple autoimmune conditions (OR, 2.35 [95% CI, 1.91-2.89]) had a higher likelihood of biologic use during pregnancy compared with those with rheumatoid arthritis. In contrast, patients with multiple sclerosis (OR, 0.19 [95% CI, 0.13-0.28]), systemic lupus erythematosus (OR, 0.29 [95% CI, 0.15-0.54]), and psoriasis or psoriatic arthritis (OR, 0.65 [95% CI, 0.52-0.80]) were less likely to use biologics during pregnancy (Figure 2). An increasing secular trend of biologic use during pregnancy from 2011 to 2021 was also noted (OR, 2.55 [95% CI, 1.80-3.62]). Similar results were observed among pregnancies with non–live birth outcomes, although the association between autoimmune disease and year of conception was less pronounced (Figure 3).

### Trends in Biologic Use During the Study Period

We observed a significant increasing trend in the proportion of pregnancies each year with at least 1 episode of biologic use (from 64.3% [330 of 513] in 2011 to 75.2% [481 of 640] in 2021; *P* < .001). When examining the type of biologic used during pregnancy over the 10-year period, we observed a significant decrease in the use of agents within the TNF inhibitor class (from 95.7% [338 of 353] in 2011 to 65.7% [357 of 543] in 2021; *P* < .05). Other biologics, including vedolizumab, abatacept, natalizumab, and belimumab, showed an increase in use, accounting for 2.3% (8 of 353) of biologic use during pregnancy in 2011 and 14.4% (78 of 543) in 2021 (eFigure 2 and eTable 5 in Supplement 1).

## Discussion

This retrospective cohort study examined biologic use during pregnancy and post partum for 7 autoimmune conditions ranging from gastrointestinal conditions (Crohn disease, ulcerative colitis), rheumatologic conditions (rheumatoid arthritis, psoriasis or psoriatic arthritis, ankylosing spondylitis, systemic lupus erythematosus), and neurologic conditions (multiple sclerosis). It is the first study to our knowledge to focus on pregnancies with preconception biologic use to capture patient and clinician decisions involving pregnancy-specific benefits and risks. The study is also the first to consider use in pregnant populations with live and non–live birth outcomes. Our study has several key findings. First, approximately one-third of patients with autoimmune conditions with prior biologic use discontinued use during pregnancy, with use declining steadily with each trimester. Second, continuation of biologics into pregnancy was highly variable across autoimmune diseases. Third, although biologic use rebounded after pregnancy, not all patients resumed biologic use post partum.

Our findings confirm the persistent pattern of biologic discontinuation during pregnancy as observed in earlier studies in which use of biologics and other immunomodulators declined in use as pregnancy progressed.^12,32,33,34^ The observed variation in discontinuation across autoimmune conditions is consistent with earlier studies that also observed a variation in the use of immunomodulators in women with various rheumatic conditions.^8,35,36^ The pattern of decline in use observed in our study may, in part, reflect relevant autoimmune disease treatment recommendations. In patients with inflammatory bowel disease (ulcerative colitis or Crohn disease), TNF inhibitors are recommended during pregnancy, with final doses administered several half-lives before the expected delivery date.^22^ For rheumatologic conditions, patients taking adalimumab, infliximab, and golimumab are conditionally recommended to continue use during the first half of pregnancy^24^ but to discontinue TNF inhibitors in the third trimester.^23^

The observed differences in the proportion of pregnancies with biologic use across autoimmune conditions may also suggest variability in improvement of the patient’s condition associated with physiologic changes during pregnancy. In patients with rheumatoid arthritis^37^ and multiple sclerosis,^38,39,40^ improvement in disease activity has been observed among pregnant patients, potentially justifying biologic discontinuation. However, in patients with ulcerative colitis, pregnancy has been associated with an increased risk for relapse,^41^ which may lead to a higher likelihood of adherence to biologics.^42^ Although up to 90% of pregnant patients with systemic lupus erythematosus have reported a relapse of their condition during pregnancy,^37,38,43^ the higher discontinuation rates observed in our study within this subpopulation suggest that other factors may contribute to biologic discontinuation, such as a stronger preference to avoid system lupus erythematosus–specific biologics (eg, belimumab) due to currently insufficient safety information^44^ or the patient achieving disease remission in which the biologic may no longer be needed. Other reasons such as the lack of pregnancy-specific evidence on the benefits and risks for agents more specifically used by this subpopulation may contribute to the decline in use.^45^ Differences in the types of biologics used to treat each autoimmune disease during pregnancy may also contribute to the likelihood for continuation as observed in the variability in classes of biologics used for each autoimmune condition (eFigure 1 in Supplement 1).

Unlike use during pregnancy, recommendations for using biologics post partum are consistently favorable, yet this does not appear to be reflected in our study in which approximately one-fourth of the patients did not resume biologic use. Postpartum use may reduce the risk of disease exacerbation often observed in the early stages after pregnancy (eg, systemic lupus erythematosis^37,38,43,46^ and multiple sclerosis^34,39^), with minimal concerns for transfer to breastmilk.^47^ Evaluation of factors contributing to avoidance of biologic use for each autoimmune condition during and after pregnancy is needed to improve our understanding of potential gaps in care.

Despite the considerable role of biologics in patients with autoimmune conditions and the relatively higher prevalence of autoimmune disease in women, comparative studies evaluating maternal, fetal, and infant safety with biologic use and the consequences of discontinuing use remain minimal. Initial studies have highlighted the importance of maintaining biologic treatment throughout the pregnancy period. In individuals with inflammatory bowel disease, discontinuation of adalimumab^48^ and infliximab^49^ during pregnancy has been associated with the risk for flares, which are associated with premature deliveries. However, studies in the pregnant population, particularly those that have evaluated recent therapies, are inherently limited by small sample sizes,^23,24,50,51^ with similar challenges in evaluating outcomes of infants exposed to biologics in utero.^52^ Furthermore, much of the current evidence on biologic use during pregnancy originates from assessments within the more prevalent conditions, such as inflammatory bowel disease,^14,17,18,21,53^ which may not be generalizable to all autoimmune conditions. Although not the primary focus of this study, our findings emphasize the need for comprehensive autoimmune disease and, ideally, indication and agent-specific evaluations of maternal, pregnancy, and infant outcomes to support informed decision-making on benefits and risks of use during pregnancy. Such studies might evaluate the effects of continued use of biologics on the complex and interdependent association between pregnancy and autoimmune disease and maternal and fetal risks.

### Limitations

Our study has several limitations. First, we measured biologic use from pharmacy dispensing claims, which indicated receipt of the drug rather than actual administration. However, we expected the likelihood of not administering the drug after receipt of the medication to be low, considering the underlying severity of the patient’s autoimmune condition and several biologics in this study requiring special pharmacy procurement processes. Second, we acknowledge the potential for misclassification of pregnant users and nonusers of biologics, particularly among whom the date of dispensing was close to our estimated date of conception. Although we used a validated pregnancy algorithm with high specificity and sensitivity, the date of conception was inferred from estimates of gestational age at pregnancy end and may not have coincided with the actual conception date.^31^ Third, our study did not examine factors other than autoimmune disease type that may have contributed to continuing biologics during pregnancy. Examples include disease severity and the types of biologics used before conception. Furthermore, due to limitations in sample size for less prevalent autoimmune conditions, specifically multiple sclerosis, ankylosing spondylitis, and system lupus erythematosus, results pertaining to these conditions should be interpreted with caution. This limitation also led to our inability to account for the effect of each therapeutic class of biologics within an autoimmune disease. Finally, our study included only privately insured individuals and may not be generalizable to all pregnant patients with autoimmune conditions in the US.

## Conclusions

In this retrospective cohort study, use of biologics during pregnancy varied depending on the patients’ autoimmune conditions, suggesting differences in perceived or true risks and benefits. Future condition-specific studies evaluating safety and efficacy of biologics are needed to enhance our understanding of the risks and benefits of biologic use for both mother and infant.

## Notes

Supplement 1. **eTable 1.** *ICD* Diagnosis Codes for Identification of Autoimmune Disease **eTable 2.** Biologics Evaluated for Use Among the Study Population **eTable 3.** Use of Biologics Across Pregnancy Periods Stratified by Indication (Live Birth Outcomes) **eTable 4.** Distribution of Biologic Use Episodes From Preconception to Postpartum Period (Live and Non–Live Birth Outcomes) **eTable 5.** Proportion of Pregnancies With Biologic Use and Episodes of Biologic Use by Therapeutic Class (2011-2021) **eFigure 1.** Therapeutic Classification of Biologics Used Anytime During Pregnancy Among Individuals With Autoimmune Disease **eFigure 2.** Trends in Use of Biologics During Pregnancy Among Patients With Conception From 2011 to 2021 (Live and Non–Live Birth Outcomes) by Autoimmune Disease and Therapeutic Class

Supplement 2.
Data Sharing Statement
