# Supplementary material for: Use of Biologics During Pregnancy Among Patients With Autoimmune Conditions
Source: JAMA Netw Open. 2025 May 15;8(5):e2510504. doi: 10.1001/jamanetworkopen.2025.10504 (PMC12082371; doi:10.1001/jamanetworkopen.2025.10504)
Supplement: Supplement 1. — eTable 1. ICD Diagnosis Codes for Identification of Autoimmune Disease eTable 2. Biologics Evaluated for Use Among the Study Population eTable 3. Use of Biologics Across Pregnancy Periods Stratified by Indication (Live Birth Outcomes) eTable 4. Distribution of Biologic Use Episodes From Preconception to Postpartum Period (Live and Non–Live Birth Outcomes) eTable 5. Proportion of Pregnancies With Biologic Use and Episodes of Biologic Use by Therapeutic Class (2011-2021) eFigure 1. Therapeutic Classification of Biologics Used Anytime During Pregnancy Among Individuals With Autoimmune Disease eFigure 2. Trends in Use of Biologics During Pregnancy Among Patients With Conception From 2011 to 2021 (Live and Non–Live Birth Outcomes) by Autoimmune Disease and Therapeutic Class [file jamanetwopen-e2510504-s001.pdf]

Supplemental Online Content

Ewig CLY, Wang Y, Smolinski NE, et al. Use of biologics during pregnancy among patients with autoimmune conditions. *JAMA Netw Open*. 2025;8(5):e2510504. doi:10.1001/jamanetworkopen.2025.10504

eTable 1. *ICD* Diagnosis Codes for Identification of Autoimmune Disease

eTable 2. Biologics Evaluated for Use Among the Study Population

eTable 3. Use of Biologics Across Pregnancy Periods Stratified by Indication (Live Birth Outcomes)

eTable 4. Distribution of Biologic Use Episodes from Preconception to Postpartum Period (Live and Non–Live Birth Outcomes)

eTable 5. Proportion of Pregnancies With Biologic Use and Episodes of Biologic Use by Therapeutic Class (2011-2021)

eFigure 1. Therapeutic Classification of Biologics Used Anytime During Pregnancy Among Individuals With Autoimmune Disease

eFigure 2. Trends in Use of Biologics During Pregnancy Among Patients With Conception From 2011 to 2021 (Live and Non–Live Birth Outcomes) by Autoimmune Disease and Therapeutic Class

This supplemental material has been provided by the authors to give readers additional information about their work.

© 2025 Ewig CLY et al. *JAMA Network Open*.

eTable 1. ***ICD*** **Diagnosis Codes for Identification of Autoimmune Disease**

	ICD-CM-9 Code		ICD-CM-10 Code
**Crohn's disease**			
Regional enteritis of small intestine	555.0	Crohn's Disease of small intestine without complications	K50.0
Regional enteritis of large intestine	555.1	Crohn's Disease of large intestine	K50.1
Regional enteritis of small intestine with large intestine	555.2	Crohn's Disease of both small and large intestine	K50.8
Regional enteritis of unspecified site	555.9	Crohn's Disease, unspecified	K50.9
**Ulcerative Colitis**			
Ulcerative (chronic) enterocolitis	556.0	Ulcerative (chronic) pancolitis	K51.0
Ulcerative (chronic) ileocolitis	556.1	Ulcerative (chronic) proctitis	K51.2
Ulcerative (chronic) proctitis	556.2	Ulcerative (chronic) rectosigmoiditis	K51.3
Ulcerative (chronic) proctosigmoiditis	556.3	Inflammatory polys of colon	K51.4
Pseudopolyposis of colon	556.4	Left sided colitis/ left hemicolitis	K51.5
Left-sided ulcerative (chronic) colitis	556.5	Other ulcerative colitis	K51.8
Universal ulcerative (chronic) colitis	556.6	Ulcerative colitis, unspecified	K51.9
Other ulcerative colitis	556.8	Other and unspecified noninfective gastroenteritis and colitis	
Ulcerative colitis unspecified	556.9	Indeterminate colitis / Colonic inflammatory bowel disease unclassified	K52.3
Other and unspecified noninfectious gastroenteritis and colitis	558.9	Other specified noninfective gastroenteritis and colitis	K52.8
		Noninfective gastroenteritis and colitis, unspecified	K52.9
**Rheumatoid Arthritis**[a]			
Rheumatoid arthritis	714.0	Rheumatoid Arthritis with rheumatoid factor	
Felty's syndrome	714.1	Felty's syndrome	M05.0

© 2025 Ewig CLY et al. *JAMA Network Open*.

Other rheumatoid arthritis with visceral or systemic involvement	714.2	Rheumatoid lung disease with rheumatoid arthritis	M05.1
Juvenile chronic polyarthritis	714.3	Rheumatoid vasculitis with rheumatoid arthritis	M05.2
Chronic postrheumatic arthropathy	714.4	Rheumatoid heart disease with rheumatoid arthritis	M05.3
Other specified inflammatory polyarthropathies	714.8	Rheumatoid myopathy with rheumatoid arthritis	M05.4
Unspecified inflammatory polyarthropathy	714.9	Rheumatoid polyneuropathy with rheumatoid arthritis	M05.5
		Rheumatoid arthritis with involvement of other organs and systems	M05.6
		Rheumatoid arthritis with rheumatoid factor without organ or systems involvement	M05.7
		Other rheumatoid arthritis with rheumatoid factor	M05.8
		Rheumatoid arthritis with rheumatoid factor, unspecified	M05.9
		Other rheumatoid arthritis	
		Rheumatoid arthritis without rheumatoid factor	M06.0
		Other specified rheumatoid arthritis	M06.8
		Rheumatoid arthritis, unspecified	M06.9
		Unspecified juvenile rheumatoid arthritis	M08.0
		Juvenile ankylosing spondylitis	M08.1
		Juvenile rheumatoid arthritis with systemic onset	M08.2
		Pauciarticular juvenile rheumatoid arthritis	M08.4
		Other juvenile arthritis	M08.8
		Juvenile arthritis, unspecified	M08.9

© 2025 Ewig CLY et al. *JAMA Network Open*.

		Other specific joint derangements	
**Ankylosing spondylitis**			
Ankylosing spondylitis	720.0	Juvenile ankylosing spondylitis	M08.1
Spondylosis and allied disorders	721.0	Ankylosing spondylitis of multiple sites in spine	M45.0
Peripheral enthesopathies and allied syndromes	726.0	Ankylosing spondylitis of occipito-atlanto-axial region	M45.1
		Ankylosing spondylitis of cervical region	M45.2
		Ankylosing spondylitis of cervicothoracic region	M45.3
		Ankylosing spondylitis of thoracic region	M45.4
		Ankylosing spondylitis of thoracolumbar region	M45.5
		Ankylosing spondylitis lumbar region	M45.6
		Ankylosing spondylitis of lumbosacral region	M45.7
		Ankylosing spondylitis sacral and sacrococcygeal region	M45.8
		Ankylosing spondylitis of unspecified sites in spine	M45.9
**Multiple Sclerosis**			
Multiple Sclerosis	340.0	Multiple Sclerosis	G35
Inflammatory diseases of the central nervous system	323.0		
Neuromyelitis Optica Spectrum Disorder	341.0		
**Psoriasis (incl psoriatic arthritis)**			
Psoriatic arthritis	696.0	Psoriasis vulgaris	L40.0
Psoriasis	696.1	Generalized pustular psoriasis	L40.1
		Acrodermatitis continua	L40.2
		Pustulosis palmaris et plantaris	L40.3

© 2025 Ewig CLY et al. *JAMA Network Open*.

		Guttae psoriasis	L40.4
		Arthropathic psoriasis	L40.5
		Other psoriasis	L40.8
		Psoriasis, unspecified	L40.9
**Systemic Lupus Erythematosus**			
Systemic lupus erythematosus	710.0	Systemic lupus erythematosus with organ or system involvement	M32.1
		Other forms of systemic lupus erythematosus	M32.8
		Systemic lupus erythematosus, unspecified	M32.9

a. includes Juvenile Idiopathic Arthritis

© 2025 Ewig CLY et al. *JAMA Network Open*.

eTable 2. Biologics Evaluated for Use Among the Study Population

Product Name[a]	Therapeutic Category
Adalimumab	Tumor Necrosis Factor (TNF)-inhibitors
Certolizumab Pegol	
Etanercept	
Golimumab	
Infliximab	
Anakinra	Interleukin (IL) Inhibitors
Brodalumab	
Canakinumab	
Guselkumab	
Ixekizumab	
Risankizumab	
Sarilumab	
Secukinumab	
Tildrakizumab	
Tocilizumab	
Ustekinumab	
Belimumab	B-cell Depleting Therapies (BCDT)
Rituximab	
Ocrelizumab	
Ofatumumab	
Abatacept	Others
Natalizumab	
Vedolizumab	
Alemtuzumab	

[a] Biosimilars if available were also included in our search.

© 2025 Ewig CLY et al. *JAMA Network Open*.

eTable 3. Use of Biologics Across Pregnancy Periods Stratified by Indication (Live Birth Outcomes)

	No. of pregnancies	1st trimester	2nd trimester	3rd trimester	Post-partum
Diagnosis[a]	N	n (%, 95% CI)	n (%, 95% CI)	n (%, 95% CI)	n (%, 95% CI)
Any AID	4342	2981 (68.7) [67.2 -70.0]	2555 (58.8) [57.3 -60.3]	2113 (48.7) [47.1 -50.1]	3350 (77.1) [75.8 -78.3]
UC	432	383 (88.6) [85.1 -91.2]	374 (86.6) [83.0 -89.4]	329 (76.1) [71.9 -79.9]	373 (86.3) [82.7 -89.2]
CD	981	877 (89.4) [87.3 -91.1]	859 (87.6) [85.3 -89.4]	709 (72.3) [69.3 -74.9]	858 (87.5) [85.2 -89.3]
RA	913	514 (56.3) [53.0 -59.4]	359 (39.3) [36.2 -42.5]	284 (31.1) [28.0 -34.0]	658 (72.0) [69.0 -74.8]
Ps/PsA	650	312 (48.0) [44.1 -51.8]	194 (29.8) [26.4 -33.4]	139 (21.4) [18.4 -24.7]	423 (65.1) [61.3 -68.6]
MS	165	49 (29.7) [23.2 -37.0]	17 (10.3) [6.5 -15.8]	12 (7.3) [4.2 -12.2]	114 (69.1) [61.6 -75.6]
AS	128	71 (55.5) [46.8 -63.7]	55 (43.0) [34.7 -51.6]	45 (35.2) [27.4 -43.7]	83 (64.8) [56.2 -72.5]
SLE	46	15 (32.6) [20.8 -47.0]	5 (10.9) [4.7 -23.0]	4 (8.7) [3.4 -20.3]	22 (47.8) [34.1 -61.8]
Multiple AID	1027	760 (74.0) [71.2 -76.5]	692 (67.4) [64.4 -70.1]	591 (57.5) [54.5 -60.5]	819 (79.7) [77.1 -82.0]

[a.] UC (Ulcerative Colitis) ; CD (Crohn's Disease) ; RA (Rheumatoid Arthritis); Ps/PsA (Psoriasis/ Psoriatic Arthritis); MS (Multiple Sclerosis); AS (Ankylosing Spondylitis); SLE (Systemic Lupus Erythematosus); Multiple AID (individuals with more than one of the aforementioned diagnosis)

© 2025 Ewig CLY et al. *JAMA Network Open*.

eTable 4. Distribution of Biologic Use Episodes From Preconception to Postpartum Period (Live and Non–Live Birth Outcomes)

	Preconception	1st trimester	2nd trimester	3rd trimester	Post-Partum
No. of utilization episodes [a], n(%)	6894	4710	2954	2419	5229
TNF- inhibitors	5353 (77.6)	3792 (80.5)	2492 (84.4)	2020 (83.5)	4360 (83.4)
Adalimumab	2166 (31.4)	1531 (32.5)	959 (32.5)	760 (31.4)	1713 (32.8)
Infliximab	1380 (20.0)	1152 (24.4)	840 (28.4)	703 (29.1)	1229 (23.5)
Etanercept	1034 (15.0)	551 (11.7)	291 (9.8)	209 (8.6)	751 (14.4)
Certolizumab	670 (9.7)	497 (10.6)	358 (12.1)	313 (12.9)	587 (11.2)
Golimumab	103 (1.5)	61 (1.3)	44 (1.5)	35 (1.4)	80 (1.5)
Interleukin inhibitors	606 (8.8)	393 (8.3)	211 (7.1)	178 (7.4)	482 (9.2)
Anakinra	8 (0.1)	6 (0.1)	4 (0.1)	3 (0.1)	8 (0.2)
Tocilizumab	84 (1.2)	53 (1.1)	17 (0.6)	15 (0.6)	65 (1.2)
Ustekinumab	299 (4.3)	222 (4.7)	149 (5.0)	124 (5.1)	243 (4.6)
Secukinumab	92 (1.3)	54 (1.1)	21 (0.7)	20 (0.8)	75 (1.4)
Ixekizumab	45 (0.6)	26 (0.6)	11 (0.4)	9 (0.4)	34 (0.7)
Risankizumab	30 (0.4)	14 (0.3)	3 (0.1)	2 (0.1)	20 (0.4)
Guselkumab	38 (0.6)	11 (0.2)	0	0	28 (0.5)
Sarilumab	8 (0.1)	6 (0.1)	5 (0.2)	4 (0.2)	8 (0.2)
Brodalumab	1 (<0.1)	1 (<0.1)	1 (<0.1)	1 (<0.1)	1 (<0.1)
Tildrakizumab	1 (<0.1)	1 (<0.1)	1 (<0.1)	1 (<0.1)	1 (<0. 1)
B-cell depleting therapies	278 (4.0)	75 (1.6)	12 (0.4)	15 (0.6)	174 (3.3)
Rituximab	92 (1.3)	15 (0.3)	5 (0.2)	6 (0.2)	56 (1.1)
Ocrelizumab	101 (1.5)	16 (0.3)	2 (0.1)	3 (0.1)	69 (1.3)
Belimumab	83 (1.2)	44 (0.9)	5 (0.2)	5 (0.2)	49 (0.9)
Ofatumumab	2 (<0.1)	0	0	1 (<0.1)	0
Others [b]	648 (9.4)	450 (9.6)	242 (8.2)	206 (8.5)	513 (9.8)
Vedolizumab	320 (4.6)	279 (5.9)	200 (6.8)	169 (7.0)	296 (5.7)
Abatacept	156 (2.3)	81 (1.7)	22 (0.7)	21 (0.9)	95 (1.8)
Natalizumab	165 (2.4)	89 (1.9)	20 (0.7)	16 (0.7)	118 (2.2)

© 2025 Ewig CLY et al. *JAMA Network Open*.

Alemtuzumab	7 (0.1)	1 (<0.1)	0	0	4 (<0.1)

[a] Number of episodes of biologic use exceeds total number of pregnancies in the study. A pregnant individual may have more than one biologic exposure during each time period.
[b] This category represents medications with alternative mechanisms of action and do not fall within any of the other therapeutic categories of biologics.

© 2025 Ewig CLY et al. *JAMA Network Open*.

eTable 5. Proportion of Pregnancies With Biologic Use and Episodes of Biologic Use by Therapeutic Class (2011- 2021)

Year of Conception	Pregnancies, n(%)		Biologic episodes occurring during pregnancy, n(%)				
	No. of pregnancies	Proportion with biologic use	No. of episodes	TNF-Inhibitors	B-cell depleting therapies	IL-Inhibitors	Others [a]
2011	513	330 (64.3)	353	338 (95.7)	2 (0.6)	5 (1.4)	8 (2.3)
2012	465	283 (60.9)	315	294 (93.3)	2 (0.6)	7 (2.2)	12 (3.8)
2013	449	310 (69.0)	358	337 (94.1)	1 (0.3)	7 (2.0)	13 (3.6)
2014	469	314 (67.0)	343	309 (90.1)	2 (0.6)	17 (5.0)	15 (4.4)
2015	490	341 (69.6)	374	330 (88.2)	1 (0.3)	19 (5.1)	24 (6.4)
2016	518	367 (70.8)	415	341 (82.2)	2 (0.5)	24 (5.8)	48 (11.6)
2017	554	401 (72.4)	470	372 (79.1)	1 (0.2)	33 (7.0)	64 (13.6)
2018	660	494 (74.8)	569	425 (74.7)	6 (1.1)	65 (11.4)	73 (12.8)
2019	701	532 (75.9)	620	476 (76.8)	7 (1.1)	64 (10.3)	73 (11.8)
2020	672	540 (80.4)	611	432 (70.7)	6 (1.0)	78 (12.8)	95 (15.5)
2021	640	481 (75.2)	543	357 (65.7)	8 (1.5)	100 (18.4)	78 (14.4)

[a] Includes vedolizumab, abatacept, natalizumab, alemtuzumab

© 2025 Ewig CLY et al. *JAMA Network Open*.

eFigure 1. Therapeutic Classification of Biologics Used Anytime During Pregnancy Among Individuals With Autoimmune Disease

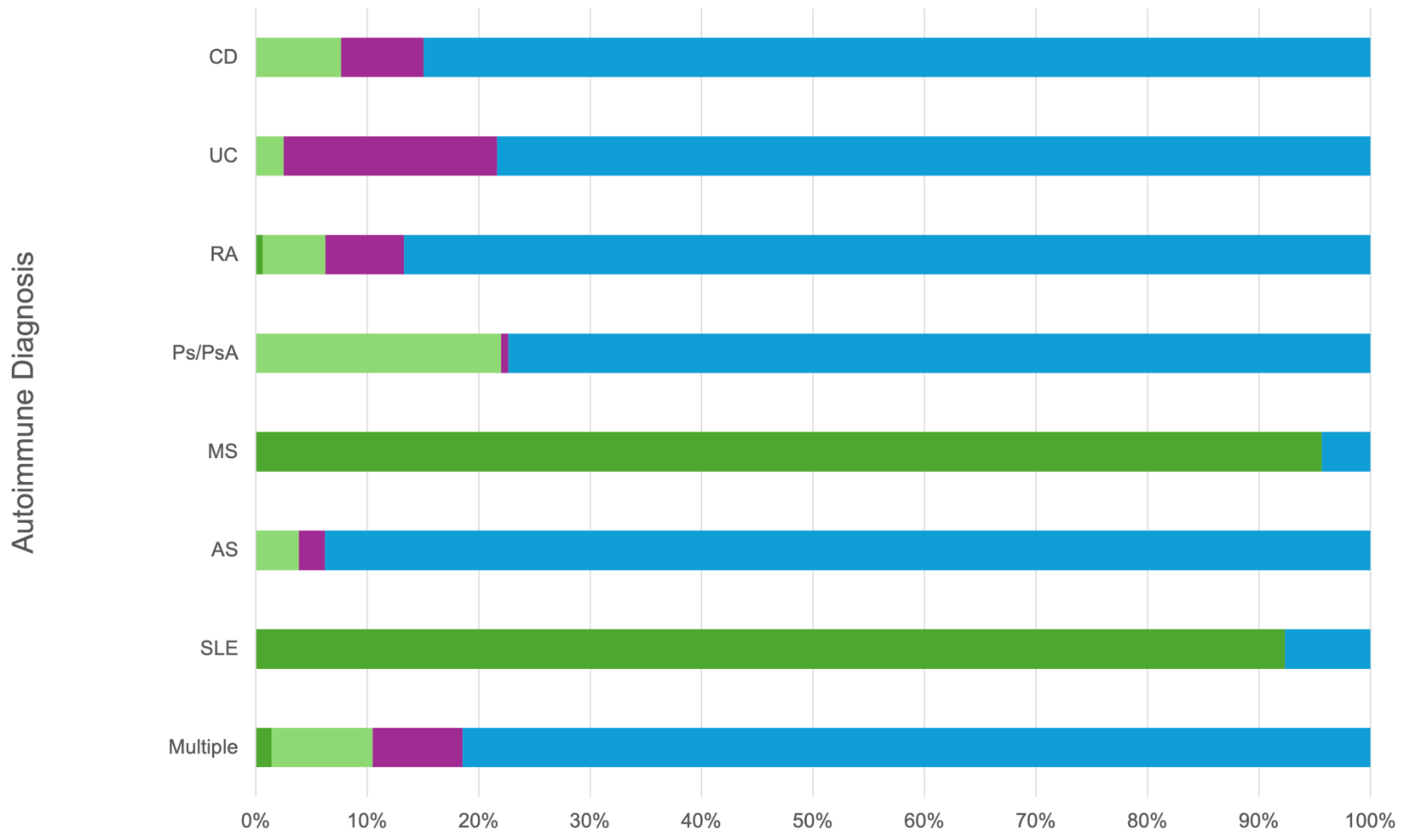

	Multiple	SLE	AS	MS	Ps/PsA	RA	UC	CD
B-cell depleting therapies	18	36	0	22	0	6	0	0
IL-Inhibitors	113	0	5	0	130	54	15	99
Others	101	0	3	0	4	68	114	96
TNF-Inhibitors	1018	3	121	1	457	835	467	1100

Autoimmune Diagnosis: UC (Ulcerative Colitis) ; CD (Crohn's Disease) ; RA (Rheumatoid Arthritis); Ps/PsA (Psoriasis/ Psoriatic Arthritis); MS (Multiple Sclerosis); AS (Ankylosing Spondylitis); SLE (Systemic Lupus Erythematosus); Multiple (individuals with more than one of the aforementioned diagnosis)

© 2025 Ewig CLY et al. *JAMA Network Open*.

eFigure 2. Trends in Use of Biologics During Pregnancy Among Patients With Conception From 2011-2021 (Live and Non–Live Birth Outcomes) by Autoimmune Disease and Therapeutic Class

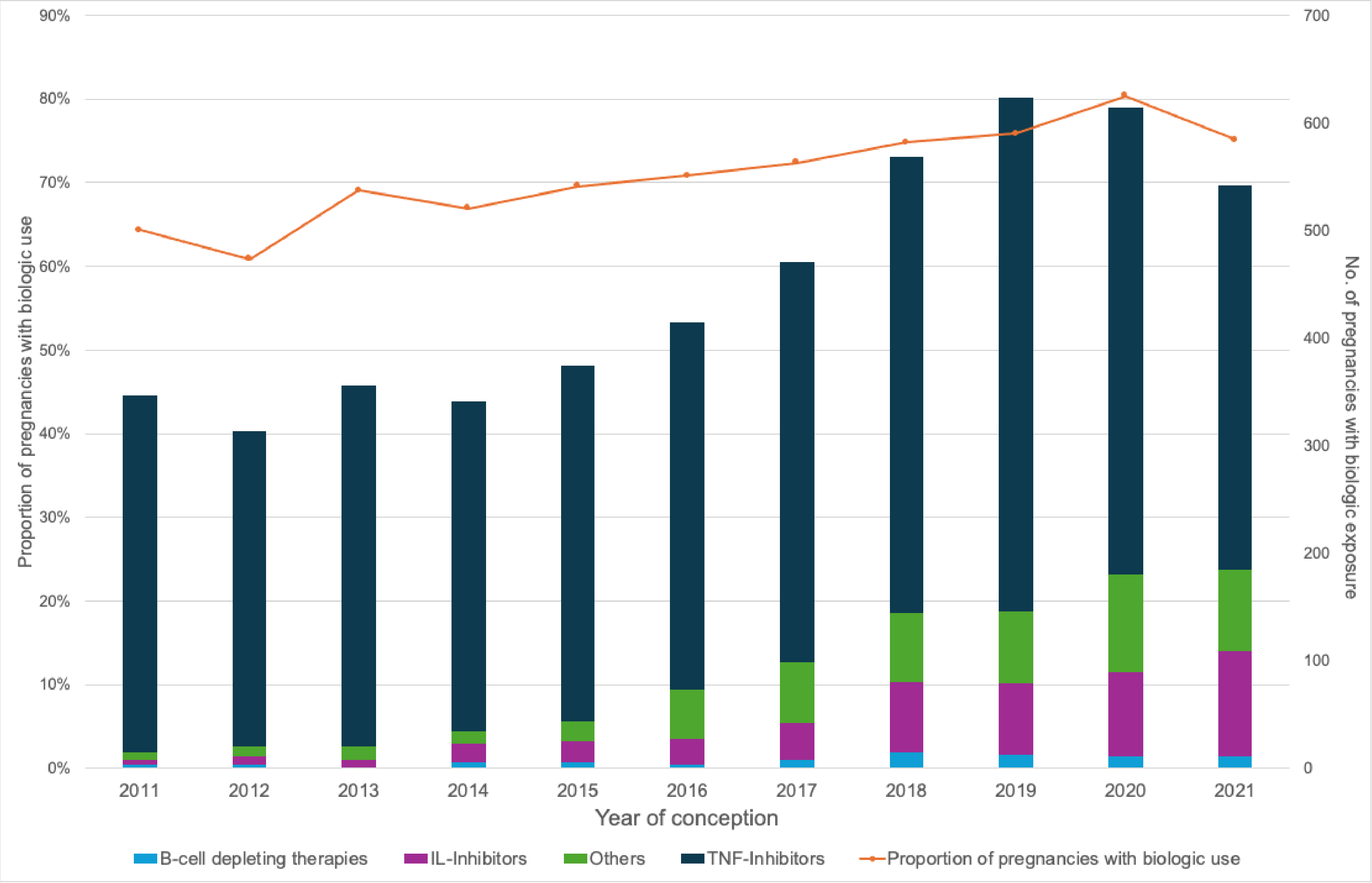

© 2025 Ewig CLY et al. *JAMA Network Open*.
