# Supplementary material for: Use of Biologics During Pregnancy Among Patients With Autoimmune Conditions
Source: JAMA Netw Open. 2025 May 15;8(5):e2510504. doi: 10.1001/jamanetworkopen.2025.10504 (PMC12082371; doi:10.1001/jamanetworkopen.2025.10504)
Supplement: Supplement 2. — Data Sharing Statement [file jamanetwopen-e2510504-s002.pdf]

Data Sharing Statement

Ewig. Use of Biologics During Pregnancy Among Patients With Autoimmune Conditions. *JAMA Netw Open*. Published May 15, 2025. doi:10.1001/jamanetworkopen.2025.10504

Data
Data available: No

Additional Information
Explanation for why data not available: The data was collected contains individual patient-level data.
